# Primary yolk sac tumor of seminal vesicle: a case report and literature review

**DOI:** 10.1186/1477-7819-10-189

**Published:** 2012-09-14

**Authors:** Xu-Dong Yao, Ya-Ping Hong, Ding-Wei Ye, Chao-Fu Wang

**Affiliations:** 1 Department of Urology, Fudan University Shanghai Cancer Center, 270 Dong’an Road, Shanghai, 200032, China; 2 Department of Oncology, Shanghai Medical College, Fudan University, Shanghai, China; 3 Department of Pathology, Fudan University Shanghai Cancer Center, Shanghai, China

**Keywords:** Yolk sac tumor, Seminal vesicle, Extragonadal

## Abstract

**Background:**

Yolk sac tumor (endodermal sinus tumor) is a rare malignant germ cell tumor arising in the testis or ovary. Extragonadal yolk sac tumor is even rarer and has only been described in case reports. Due to the rarity of the tumors, the appropriately optimal treatment remains unclear. We report a case of yolk sac tumor in the seminal vesicle.

**Case:**

A 38-year-old Asian male presented with gross hematuria and hemospermia. Transrectal ultrasound scan showed a solid mass in the left seminal vesicle and the scrotal sonography showed no abnormalities. Bilateral seminal vesicles were resected, and histopathological examination showed a typical pattern of yolk sac tumor (YST). The patient responded poorly to comprehensive treatment of radiotherapy, chemotherapy and surgeries, developed systemic multiple metastases, and died of cachexia one and half years after diagnosis.

## Background

Yolk sac tumor (YST, also called endodermal sinus tumor) most frequently occurs in the gonads and has a poor prognosis, if not treated aggressively [1].

Extragonadal yolk sac tumor (YST) is rare, and the most common sites of origin are, in order of frequency, the mediastinum, the retroperitoneum, the sacrococcygeal region and the pineal gland [2]. Due to the rarity of this tumor, the reported cases were treated by varied surgeries with or without adjuvant therapy. Thus, the optimal treatment remains unclear. To add to the literature, we report a case of yolk sac tumor originating in the left seminal vesicle.

## Case presentation

A 38-year-old Asian male presented to his urologist with gross hematuria and hemospermia of a half-year duration. The remainder of his physical examination, medical history and surgical history were unremarkable. He had no history of cryptorchidism, testicular swelling or testicular atrophy, and no family history of testicular cancer. A transrectal ultrasound scan performed on the day of presentation showed a solid mass of the left seminal vesicle. A puncture biopsy of the left seminal vesicle confirmed diagnosis of germ cell tumor. No tumorous lesions were found in other sites. At presentation, levels of serum tumor markers were obtained: α-fetoprotein (AFP) was found to be elevated to 1,740.9 ug/l (normal less than 10 ng/ml); serum β-human chorionic gonadotropin (β-hCG) (normal less than 10 mIU/ml) and prostate-specific antigen (PSA) (normal less than 4.0 ng/ml) were within normal range. The patient was given four cycles of chemotherapy consisting of bleomycin (15 mg D-2, 9, and 16), cisplatin (30 mg/m^2^ for four consecutive days), and etoposide (100 mg/m^2^ for four consecutive days). After the chemotherapy, serum AFP was found to be decreased to 58.09 ug/l, and the symptom of hemospermia disappeared.

**Figure 1:**
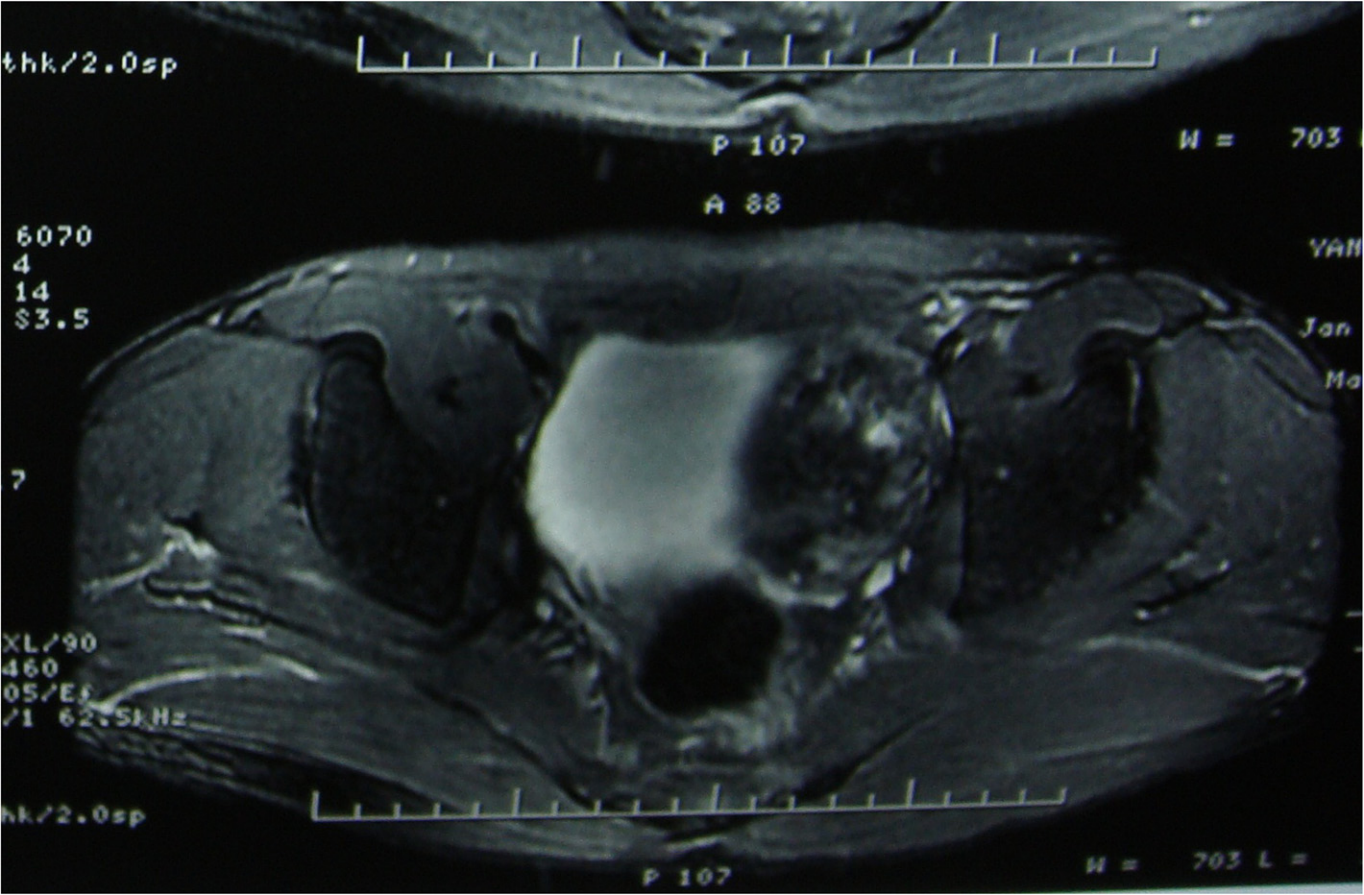
Magnetic resonance imaging (MRI): an abnormal signal mass in the left seminal vesicle.

**Figure 2:**
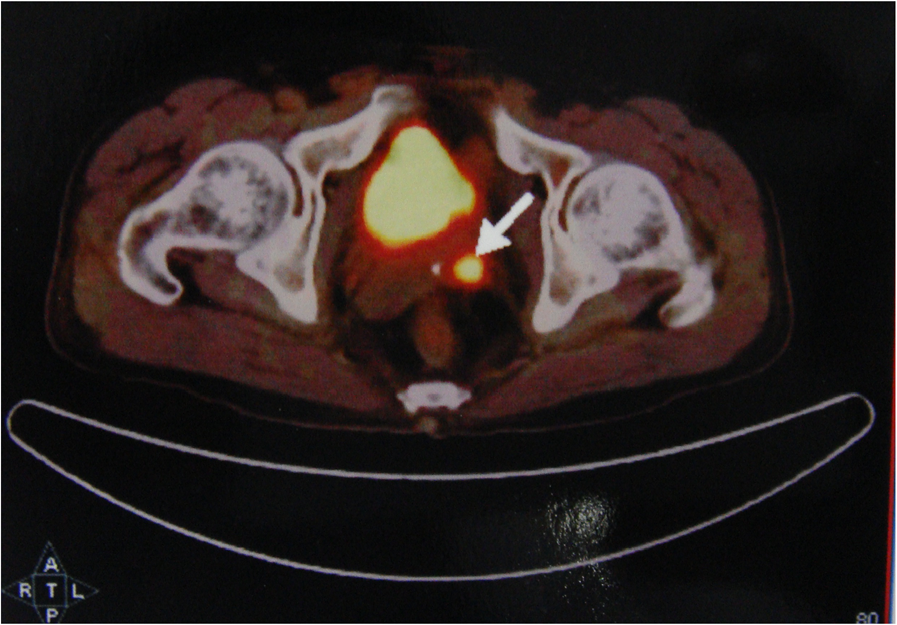
**Positron emission tomography/computed tomography (PET/CT).** There was still tumor activity in the left seminal vesicle.

**Figure 3:**
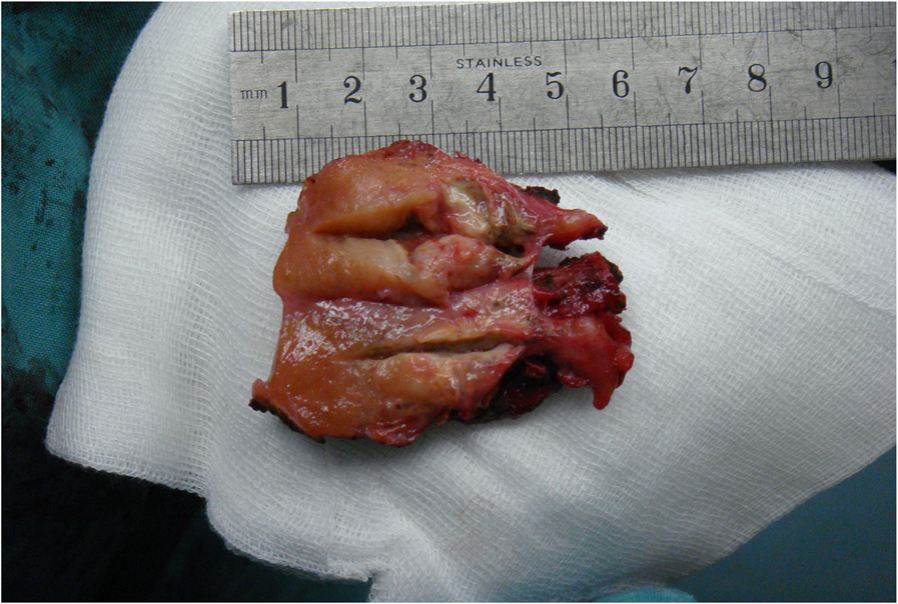
The resected specimen with the tumor showing capsulated appearance and cut surface cystic, fish-like changes.

The patient was then transferred to our hospital. He had normal testes on palpation. And a scrotal sonography was performed and revealed no abnormalities. Magnetic resonance imaging (MRI) reported an abnormal signal mass in the left seminal vesicle (Figure 1). Positron emission tomography/computed tomography (PET/CT) showed tumor activity in the left seminal vesicle (Figure 2). Extensive examination for metastases, including chest X-ray film and abdominal computed tomography (CT) showed no abnormalities. Resection of the bilateral seminal vesicles was performed. The left seminal vesicle was obviously swollen (4. × 3.0 × 2.5 cm) and was adhering widely to its surroundings. The resected tumor was a solid encapsulated mass, and the cut surface showed cystic fish-like changes (Figure 3). Although the right seminal vesicle was slightly swollen, there was no sign of tumor involvement.

**Figure 4:**
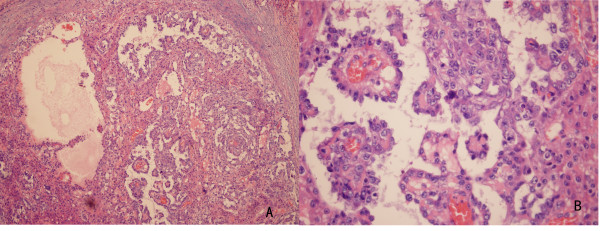
**Histopathology of the resected specimen.A**. The resected specimen was a malignant tumor composed of neoplastic cells proliferating in a microcystic or reticular growth pattern. **B**. Neoplastic cells with highly atypical middle-or large-sized nuclei and Schiller-Duval bodies partially mimicking papillary structures

**Figure 5:**
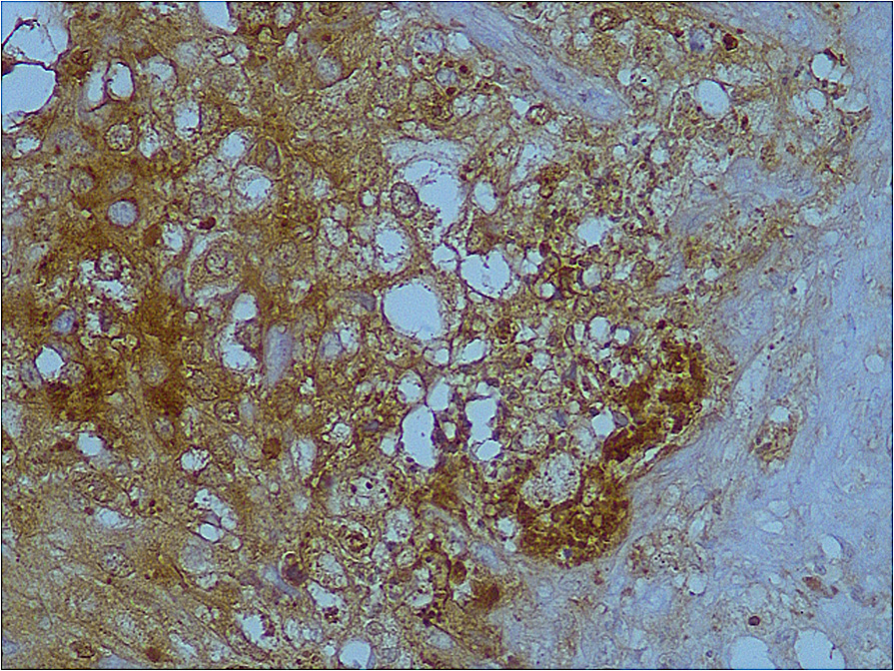
Immunohistochemical stain for alpha-fetoprotein was positive

Histological evaluation of the resected specimen exhibited a malignant tumor composed of neoplastic cells proliferating in a microcystic or reticular pattern of growth. The neoplastic cells had highly atypical middle- or large-sized nuclei, with Schiller-Duval bodies partially mimicking papillary structures (Figure 4). Immunohistochemical stain for α-fetoprotein was positive (Figure 5). Histological features of the resected specimen were interpreted as a yolk sac tumor. The cutting edge of the left seminal vesicle was not involved. Plus, the right seminal vesicle was free of the tumor. Therefore, we diagnosed his disease as a yolk sac tumor originating in the left seminal vesicle. After surgery, serum AFP decreased to 29.7 ug/l, yet it increased to 79.43 ug/l at four weeks. After the surgery, the patient received four cycles of chemotherapy consisting of bleomycin (30 U D-2, 9, and 16), cisplatin (30 mg/m^2^ for five consecutive days), and etoposide (100 mg/m^2^ for five consecutive days). On completion of the chemotherapy, serum AFP was found to have decreased to 53.24 ug/l and remained stable. However, at week 6 after chemotherapy, serum AFP again elevated to 184.9 ug/ml. Positron emission tomography (PET) of the pelvis revealed lymph node metastases in the region of the left iliac vessels. Thus, resection of the pelvic lymph nodes was performed. Enlarged lymph nodes were not found in the areas of the external iliac, internal iliac and obturator. The left side of the prostate was fixed to the pelvic wall. Histological evaluation of the specimens exhibited proliferation in the left obturator lymph tissues but showed no evidence of lymphatic metastasis. Fibrous connective tissue outside the bladder was infiltrated by lymphocytes.

On the postoperative Day 3, adjunct radiotherapy was introduced, but the response was not good, with AFP remaining at a high level since then. The patient presented rapid multiple systemic metastases and died of cachexia one and half years after diagnosis.

## Discussion

YST is a malignant germ cell tumor that usually arises in the gonads and is characteristically accompanied by an elevation of the serum α-fetoprotein level. YST is rarely found extragonadally. Case reports have described the occurrences of this entity in the pineal gland, head and neck, mediastinum, lung, stomach, liver, omentum, retroperitoneum, prostate, intrarenal, vagina, vulva, urachus and presacral regions [2-6].

The typical morphologic structures of endodermal sinus pattern known as sinuses of Duval, Schiller-Duval bodies, or glomerulus-like structures, superficially resemble immature renal glomeruli [7]. The three sensitive diagnostic markers for yolk sac tumor are alpha-fetoprotein, glypican-3 and SALL4 [3,7].

The histogenesis of extragonadal YST remains controversial. These tumors appear to arise from germ cells misplaced during embryogenesis, and malignant transformation leads to primary germ cell tumors at extragonadal sites [8].

Due to the rarity of the extragonadal YST, there is no detailed description of its epidemiology and its clinical features in particular. Additionally, due to the particularly aggressive nature of YST, the information derived from germ cell tumor (GCT) studies may have limited applicability. From the case reports we have, the age distribution of extragonadal YST, similar to that of the testicular tumors, appears to be bimodal with an early peak in early infancy and a later peak in adolescence. YST may grow to a large size with no or relatively few symptoms, and the symptoms may vary due to origin sites. The majority of YSTs produce high levels of AFP, which helps in monitoring the response to the treatment. In the past, YSTs were nearly uniformly fatal, regardless of the primary location. However, the survival rate of YST patients has been significantly improved because of the application of Cisplatin-based multiagent chemotherapy [1-6,9].

In our case, the patient had no history of swollen testes or testicular atrophy and had normal testes on palpation.Also, a scrotal sonography was performed and revealed no abnormalities in either side of the testes. With spatial resolution boosted by a high-frequency transducer, a testicular tumor may be nonpalpable but still visible sonographically [10,11]. Sonographic findings range from echogenic foci or hypoechoic areas and heterogeneous lesions to microlithiasis and macrocalcifications [11,12]. It should be noted that normal sonographic findings were more frequent before 1990 [11]. Therefore, we did not consider the diagnosis of the primary testicular tumor regressed with metastasis in the seminal vesicle. However, since the testes were not resected, we could not completely exclude this possibility without histological evaluation.

A correct diagnosis was made as late as half a year after the symptoms appeared due to the unspecific symptoms (gross hematuria and hemospermia) and rarity of the site of extragonadal YST (the seminal vesicle). This may be one of the contributors to the poor prognosis. From the clinical courses of this patient, we can see that the patient’s general response to the treatment is not very good as compared with recent reports. Jay P. Shah [13] reported a five-year survival rate of 65%. The possible reasons for the poor prognosis of our patient were the advanced age, the race and the site of the original tumor. The median age at the time of diagnosis of YSTs is 18, occurrence after 30 is exceptional [14], and prognosis in the infancy cohort is better than that in the adolescent cohorts. The age of our patient (38) may be a contributor to the poor diagnosis. In Jay P. Shah’s report, African-Americans had poorer survival compared with Caucasians. Yu-juwei Hse [15] reported the treatment results in Chinese patents with nonseminomatous extragonadal germ cell tumor (NSEGCT) are not as good as in patients in Western countries. Saxman *et al.*[16] reported the experience with chemotherapy in Indians with NSEGCT. Only 5 of 73 patients (7%) were long-term disease-free survivors after chemotherapy. More investigations need to be done to determine whether race is one of the contributors to the poor prognosis of EGCT. Several reports showed that EGCT in mediastina has a worse outcome than EGCT in other sites [15-17]. To our knowledge, there is no report about YST primarily arising in the seminal vesicle. The seminal vesicle as the site of origin contributes to the patient’s poor prognosis.

## Conclusions

We present a rare case of yolk sac tumor originating in the left seminal vesicle. Extragonadal YST is an aggressive germ cell tumor, and the treatment results are still unsatisfactory. More investigations are warranted to define the optimal treatment.

## Consent

Written informed consent was obtained from the patient’s elder brother for publication of this case report and any accompanying images. A copy of the written consent is available for review by the Editor-in-Chief of this journal.

## Abbreviations

YST: yolk sac tumor; AFP: α-fetoprotein; CT: computed tomography; MRI: magnetic resonance imaging; PSA: prostate-specific antigen; PET/CT: Positron emission tomography/computed tomography; GCT: germ cell tumor; EGCT: extragonadal germ cell tumor; NSEGCT: nonseminomatous extragonadal germ cell tumor.

## Competing interests

The authors declare that they have no competing interests.

## Authors’ contributions

XDY and HYP conceived the concept for the study, participated in drafting the manuscript, and conducted a critical review. Both authors contributed equally to this work. DWY participated in revising the manuscript critically for important intellectual content. CFW prepared the histological figures. All authors read and approved the final manuscript.

## References

[B1] Bosl GJ Motzer RJ Testicular germ-cell cancer N Engl J Med 1997 337 242 253 Erratum in: N Engl J Med 1997, 337:1403 10.1056/NEJM199707243370406 9227931

[B2] Toner GC Geller NL Lin SY Bosl GJ Extragonadal and poor risk nonseminomatous germ cell tumors Survival and prognostic features. Cancer 1991 67 2049 2057 10.1002/1097-0142(19910415)67:8<2049::aid-cncr2820670807>3.0.co;2-h 1848473

[B3] Mei K Liu A Allan RW Wang P Lane Z Abel TW Wei L Cheng H Guo S Peng Y Rakheja D Wang M Ma J Rodriguez MM Li J Cao D Diagnostic utility of SALL4 in primary germ cell tumors of the central nervous system: a study of 77 cases Mod Pathol 2009 22 1628 1636 10.1038/modpathol.2009.148 19820689

[B4] Saxman SB Nichols CR Einhorn LH Salvage chemotherapy in patients with extragonadal nonseminomatous germ cell tumors: the Indiana University experience J Clin Oncol 1994 12 1390 1393 8021729 10.1200/JCO.1994.12.7.1390

[B5] Cao D Liu A Wang F Allan RW Mei K Peng Y Du J Guo S Abel TW Lane Z Ma J Rodriguez M Akhi S Dehiya N Li J RNA-binding protein LIN28 is a marker for primary extragonadal germ cell tumors: an immunohistochemical study of 131 cases Mod Pathol 2011 24 288 296 10.1038/modpathol.2010.195 21057460

[B6] Gupta R Mathur SR Arora VK Sharma SG Cytologic features of extragonadal germ cell tumors: a study of 88 cases with aspiration cytology Cancer 2008 114 504 511 10.1002/cncr.23983 18980289

[B7] Talerman A Kurman RJ Germ cell tumors of the ovary Blaustein's Pathology of the Female Genital Tract 2002 5 New York: Springer-Verlag 967 1033

[B8] Albers P Düsseldorf Parsons KF10th edition of the European Association of Urology Guidelines 2010 The European Association of Urology

[B9] Bukowski RM Wolf M Kulander BG Montie J Crawford ED Blumenstein B Alternating combination chemotherapy in patients with extragonadal germ cell tumors. A Southwest Oncology Group study Cancer 1993 71 2631 2638 10.1002/1097-0142(19930415)71:8<2631::AID-CNCR2820710831>3.0.CO;2-G 7680950

[B10] Prim J Spontanheilung eines bösartigen wahrschein-lich chorionepitheliomatosen: gewaches im hoden Arch Pathol Anat 1927 265 239 241 10.1007/BF01894164

[B11] Tasu J-P Faye N Eschwege P Rocher L Bléry M Imaging of burned-out testis tumor: five new cases and review of the literature J Ultrasound Med 2003 22 515 521 12751863 10.7863/jum.2003.22.5.515

[B12] Kebapci M Can C Isiksoy S Aslan O Oner U Burned-out tumor of the testis presenting as supraclavicular lymphadenopathy Eur Radiol 2002 12 371 373 10.1007/s003300101038 11870436

[B13] Shah JP Kumar S Bryant CS Ali-Fehmi R Malone JM Jr Deppe G Morris RT A population-based analysis of 788 of yolk sac tumors: a comparison of males and females Int J Cancer 2008 123 2671 2675 10.1002/ijc.23792 18767035

[B14] Robova H Rob L Hrehorcak M Zoban P Prusa R Endodermal sinus tumor diagnosed in pregnancy: a case report Int J Gynecol Cancer 2007 17 914 933 10.1111/j.1525-1438.2007.00850.x 17635618

[B15] Hse Y-J Pai L Chen YC Ho CL Kao WY Chao TY Extragonadal germ cell tumor in Taiwan: an analysis of treatment results of 59 patients Cancer 2002 95 766 774 10.1002/cncr.10738 12209720

[B16] Bokemeyer C Hartmann JT Fossa SD Droz JP Schmol HJ Horwich A Gerl A Beyer J Pont J Kanz L Nichols CR Einhorn L Extragonadal germ cell tumors: relation to testicular neoplasia and management options APMIS 2003 111 49 59 discussion 59–63 10.1034/j.1600-0463.2003.11101081.x 12752235

[B17] Rosti G De Giorgi U Wandt H Lioure B Leyvraz S Kolbe K Papiani G Ballardini M Kulekci A Demirer T Solid Tumours Working Party First-line high-dose chemotherapy for patients with poor prognosis extragonadal germ cell tumors: the experience of the European Bone Marrow Transplantation (EBMT) Solid Tumors Working Party Bone Marrow Transplant 2004 34 1033 1037 10.1038/sj.bmt.1704704 15516940

